# Laser-induced fluorescence (LIF) as a smart method for fast environmental virological analyses: validation on Picornaviruses

**DOI:** 10.1038/s41598-019-49005-3

**Published:** 2019-08-29

**Authors:** Valentina Gabbarini, Riccardo Rossi, Jean-François Ciparisse, Andrea Malizia, Andrea Divizia, Patrizia De Filippis, Maurizio Anselmi, Mariachiara Carestia, Leonardo Palombi, Maurizio Divizia, Pasqualino Gaudio

**Affiliations:** 1 Department of Industrial Engineering, University of Rome “Tor Vergata”, Via del Politecnico 1, Rome, 00133 Italy; 2 Department of Biomedicine and Prevention, University of Rome “Tor Vergata”, Via di Montpellier 1, Rome, 00133 Italy

**Keywords:** Applied microbiology, Applied microbiology, Fluorescence spectroscopy, Fluorescence spectroscopy

## Abstract

Virological analysis is time-consuming and expensive. The aim of this work is to demonstrate the applicability of laser-induced fluorescence (LIF) to the classification of viruses, reducing the time for this analysis and its costs. Experimental tests were performed in which different viruses were irradiated with a UV laser emitting at 266 nm and the emitted spectra were recorded by a spectrometer. The classification techniques show the possibility of discriminating viruses. Although the application of the LIF technique to biological agents has been thoroughly studied by many researchers over the years, this work aims at validating for the first time its applicability to virological analyses. The development of a fast virological analysis may revolutionize this field, allowing fast responses to epidemiologic events, reducing their risks and improving the efficiency of monitoring environments. Moreover, a cost reduction may lead to an increase in the monitoring frequency, with an obvious enhancement of safety and prevention.

## Introduction

Virological analysis is the set of techniques used to determine the presence of specific viruses in different types of samples, from human to environmental monitoring. In this latest case, the virus analysis consists of two phases: the concentration step and the analysis of the sample. Once the volume to be analysed has been reduced, the test phase can be carried out by virus isolation on a susceptible cell line or through innovative molecular methods based on PCR, including qRT-PCR^1^, RT-PCR combined with the hybridization to specific probes^2^, nanofluidic real-time RT-PCR, digital PCR, etc.^3–5^. All these approaches show several limitations related to the nature of the viruses and the environment where they have been collected. At first, a unique cell line for the isolation does not exist, but it depends on the virus behaviour. Furthermore, some viruses do not multiply on cell lines^6,7^. The molecular methods require a small volume of sample, and the presence of other compounds may interfere with the reaction. Thus, specific primers are used, and the resulting amplified region must be sequenced and analysed by means of databases^8^.

Because these methods are time-consuming, the environmental monitoring of viruses is usually not performed. For instance, the isolation of wild-type hepatitis A virus on specific cell lines (if the particular strain can be cultured, for instance, wt HM-175 strain^9^) may take 3–5 weeks and at least two passages on cells, whereas the molecular methods require 3–5 days^10^. For this reason, drinking water analyses provide biological (bacteria) and chemical details, while virological analysis is avoided until an outbreak spreads. Furthermore, due to the long time needed, the virological analysis does not comply with proper water monitoring^11,12^.

The possibility of performing analyses in just a few minutes or even continuously is certainly an advantage if compared to the 3–5 days needed by molecular methods or the few weeks for virus isolation on cell lines. For example, consider how fast detection may be relevant in cases of virus diffusion, as it would enable countermeasures to be taken immediately.

The aim of this work is to demonstrate the applicability of laser-induced fluorescence (LIF) for virological analyses of environmental samples. By irradiating the sample with UV light, some molecules absorb and re-emit less energetic radiation (visible region). This phenomenon is called fluorescence, and the spectrum emitted is a function of the specific molecules compounding the microorganisms^13,14^. Although this technique has already been used for bacterial classification^15–22^, no evidence of its applicability to viruses has been reported in the literature.

LIF could be a proper approach to developing an instrument suitable for analysing and classifying viruses. Due to their molecular composition, different viruses emit dissimilar spectra as a fingerprint, allowing their classification. Moreover, the evaluation of the sample concentration could be performed due to the correlation of this parameter with the fluorescence intensity.

## Results

### Classification

**Figure 1:**
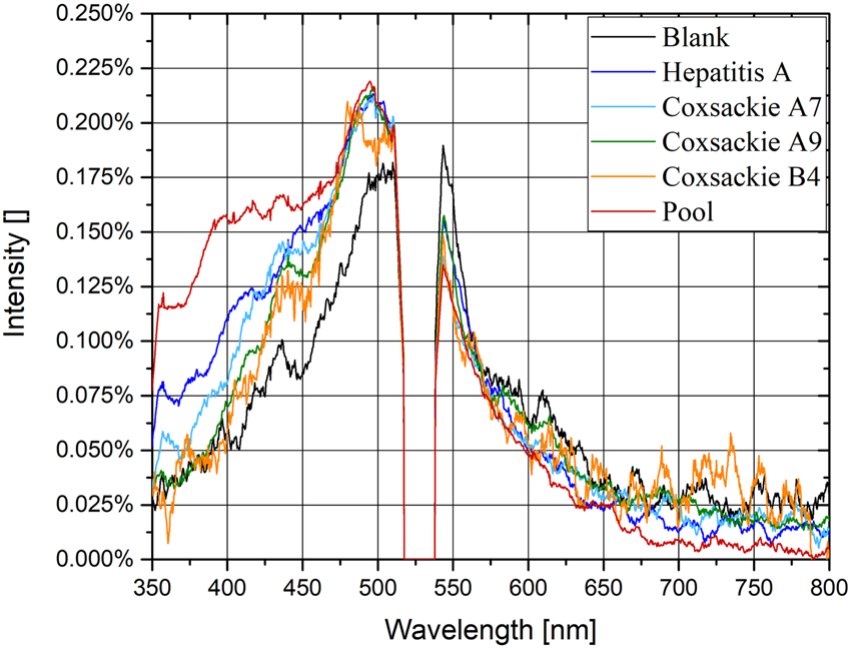
Fluorescence spectra of each sample irradiated at a wavelength of 266 nm. The exposure time of the spectrometer is 60 s. Spectra are normalised with respect to the total fluorescence intensity and calculated as the average of ten measurements.

Figure 1 shows the fluorescence spectrum of each sample when irradiated with the 266 nm laser light. The samples are the control (blank, Dulbecco completed phosphate buffered saline (PBS)), Hepatitis A, Coxsackie A7, Coxsackie A9, Coxsackie B4 and a pool of four different viruses: Rotavirus, Hepatitis A virus, Echovirus type 1, and Astrovirus. The fluorescence light ranges from 350 nm to 700 nm (after 700 nm, only noise is visible). The gap at 532 nm is due to the notch filter of the second harmonic elastic scattering (λ = 532 nm). The spectral intensity, measured by the spectrometer in counts, is adimensionalized with respect to the total fluorescence intensity. Each spectrum is the average spectrum of ten measurements. The exposure time is 60 s, and thus, each measurement is the average of 6 10^5^ laser shots (the laser pulse repetition frequency, PRF, is 10 kHz).

**Figure 2:**
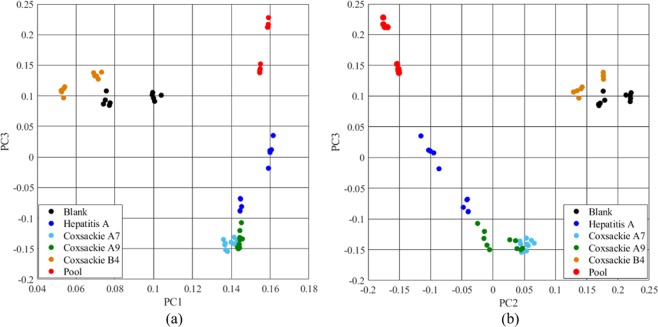
Grouped scatter plot of each sample with PC1 and PC3 (**a**) and PC2 and PC3 (**b**). Points are classified according to the different viruses analysed. Mostly good separation of each group is shown (except for Coxsackie A7 and A9, where worse separation is obtained).

Figure 2 shows the grouped scatter plot of each measurement after the application of principal component analysis (PCA)^23^. Each sample is indicated by a specific colour. Figure 2(a) is generated by Principal Component (PC) 1 and 3 (PC1 and PC3), while Fig. 2(b) exploits PC2 and PC3. Each group is well separated by the others, meaning that the spectra are dissimilar and the classification can be performed. The Coxsackie A7 and Coxsackie A9 groups are closed, and some points overlap as expected, as they belong to the same subgroup (Coxsackie A) of the family *Picornaviridae* and the chemical composition is assumed to be similar, although Coxsackie A7 belongs to the Enterovirus genus A and Coxsackie A9 to the Enterovirus genus B. The Coxsackie B4 group is near the blank spectrum. In fact, due to the signal with a small signal-to-noise ratio (SNR) (with the signal highly noised), the fluorescence measured in the case of Coxsackie B4 may be confused with the blank sample. A future improvement of the experimental setup to increase its sensitivity (time exposure, laser intensity, etc.) may lead to a better separation.

**Figure 3:**
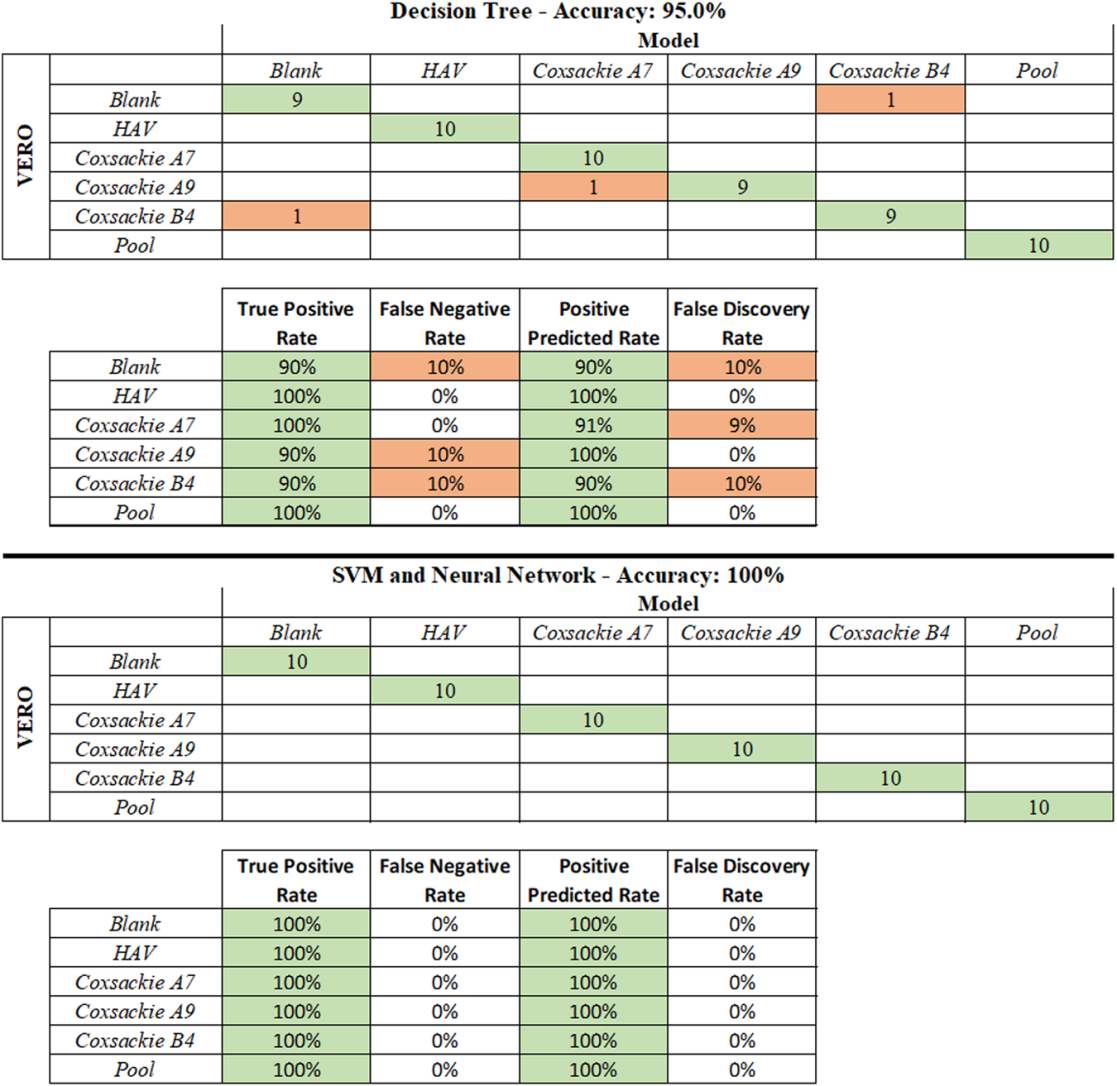
Confusion matrix with the true positive rate, false negative rate, positive predicted rate and false discovery rate, with two different classification algorithms applied: the decision tree and the support vector machine (SVM) and neural network.

Figure 3 shows the confusion matrix obtained using three classification algorithms: the decision tree, the support vector machine (SVM) and the neural network^24–26^. All the classification results are obtained using only the first three principal components. The decision tree missed the classification three times. It confused the blank spectrum with Coxsackie B4 and *vice versa*, probably due to the low intensity of the latter. The third mistake was made between Coxsackie A7 and A9. In this case, similar spectra are expected due to the similarity between the two serotypes of the same group of viruses. The true positive rate was 90% for the blank sample and for Coxsackie A9 and B4, whereas it was 100% for the others. The positive predicted rate was 90% for the blank and Coxsackie B4 samples, 91% for Coxsackie A7, and 100% for the others. The overall accuracy of the classification was 95%. The SVM and the neural network gave the best results, with a perfect classification of each sample. The positive predicted rate, the true positive rate and the overall accuracy were 100%. The neural network was tested both with and without the PCA pre-processing, and the results were always the same.

### Sensitivity

**Figure 4:**
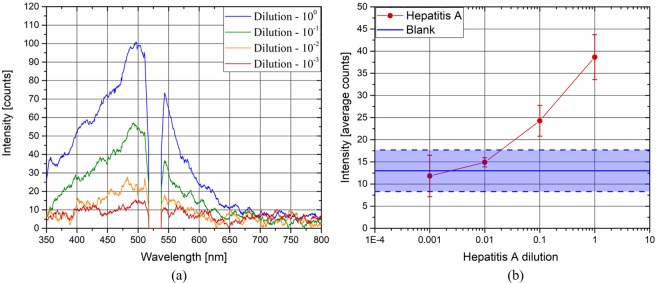
Hepatitis A spectrum at each concentration (**a**), and average fluorescence intensity of Hepatitis A and blank samples as a function of the concentration of the virus (**b**).

Figure 4 shows the correlation between the Hepatitis A concentration and fluorescence intensity. Figure 4(a) shows the average spectra of the virus at concentrations from 2 10^6^ to 2 10^3^. The larger the concentration of the virus, the more intense the fluorescence light is. It is remarkable that the shapes of the spectra acquired are similar, especially for the higher concentrations (2 10^6^ TCID50/mL and 2 10^5^ TCID50/mL). When the concentration is decreased, the shape of the spectrum seems to change. However, this effect is due to the decrease in the SNR, which involves a noisier spectrum. Figure 4(b) shows the average total fluorescence intensity (red dots) as a function of the virus concentration. The blue line represents the average blank fluorescence intensity, whereas the blue area represents the error band of its value. Note that the intensities of the lower concentrations overlap with the blank intensities. Therefore, the sensitivity of the concentration measurement of Hepatitis A with the current experimental apparatus is limited between 2 10^4^ and 2 10^5^ TCID50/mL. However, a large increase in the sensitivity may be achieved by improving the experimental apparatus, as described in the next section.

## Discussion

In this work, the measurement of laser-induced fluorescence on viruses has been investigated, and the results show the interesting potential of this technique. Basically, it has been demonstrated that the classification of viruses could be performed by means of this technique. The LIF approach is based on exciting the fluorophores contained in the envelope proteins of the viruses by proper radiation (in our case, the wavelength was 266 nm). Since differences between the chemical compositions of viruses are expected, different fluorescence spectra are expected as well. Thus, the fluorescence spectrum is characteristic of the viruses shown, similar to a fingerprint. By developing a database where the spectrum of each virus is recorded, it could be feasible to classify the virus analysed accordingly. The classification is allowed by a proper classification algorithm, such as the three shown in this work (decision tree, support vector machine and neural network). In our simplified case, where the number of classes is six, this algorithm allowed us to reach a very high accuracy (95%, 100% and 100%). In a real case, where the number of possibilities may be much greater, these algorithms and this approach should be integrated with more sophisticated machine learning methods. Furthermore, much interference may occur due to the presence of other agents (e.g., chemicals, bacteria, or fungi). The environmental viruses available in the aqueous and food samples belong to 7–8 species. As all of them show the same behaviour, it is not unlikely that two or more viruses can be present at the same time. Isolating and classifying two or more viruses from the same sample is not simple neither with cellular lines nor with molecular methods. In this case, the pool of four viruses was well distinguished from the single viruses. To date, it is practically impossible to discriminate the different viruses in the pool. However, the classification of different strains is theoretically possible. For the bacterial classification, data scientists are working to find the best algorithm to classify different agents in a pool. However, this evaluation requires optimization of the spectra, which may be achieved by increasing the power of the laser, the time of lamp exposure and the number of measurements. Thus, the technology used to carry out the tests is still not optimized and needs to be improved to be applicable in real contexts. First, the classification of a larger number of viruses may be solved by increasing the number of wavelengths that irradiate the sample. Basically, the fluorescence spectrum changes with the irradiation wavelength, as the fluorescence cross-section changes with the wavelength, and some fluorophores become dominant only at specific wavelengths^15^. Thus, it could be viable to obtain more “fingerprints” for each virus, involving a larger amount of information and a more accurate classification. For the interference, different solutions should be investigated. First, the spectra of interference commonly found in the specific application may be recorded. Thus, the classifier algorithm may also be trained with the fluorescence spectra of the background material. However, its huge variability may increase the uncertainties of the classification. Alternatively, a middle step, such as a differential centrifugation of the sample, may help to exclude some of these uncertainties. Finally, the classification of a virus in a mixture of viruses should be theoretically achievable, as the fluorescence spectrum of the sample should be the sum of the fluorescence emitted by each virus.

The sensitivity analysis, carried out by diluting Hepatitis A, has shown that the system is able to perform a measurement with a small concentration of viruses. In our case, the lower limit is between 2 10^4^ and 2 10^5^ TCID50/mL. Furthermore, the system should be improved, allowing a measurement of smaller concentrations. For example, the spectrometer used has a maximum exposure time of 1 minute. If the exposure time is increased to 10 minutes, the minimum readable concentration may be 10 times lower if no linear effects are observed (no linear effects may arise only in the case of large concentrations, which is not the case here). The fluorescence light may also be increased by intensifying the laser radiation. The collecting system efficiency may also be improved to increase the amount of fluorescence light analysed.

It is good to remember that the molecular tests at the beginning had low sensitivity. The sensitivity was increased by using the new generation kits, purifying the samples, improving the primer sets, etc.

In conclusion, it is believed that the LIF method may be the basis for the development of a new tool to analyse and monitor viruses. The main advantage of this technique concerns the time of the analysis, considering that this measurement should require an analysis time no longer than a few hours (for very small virus concentration). Furthermore, this tool may involve a significant reduction in the costs of virus analysis, as the fixed costs should be high but the variable costs (i.e., electricity and manpower) should be near zero.

### Potential and future opportunities

This method may find applications in many fields, e.g., the continuous monitoring of the environment, such as the drinking water. It may be possible to develop a stand-off instrument able to detect and classify the presence of specific harmful viruses and to give a proper alarm. It may also be linked with a disinfection system that intervenes only when needed, avoiding unnecessary operations and reducing energy consumption and costs. For the bacterial classification in the literature, the technology may be used as a point sensor or a remote range apparatus^15^. The same approach may be used to develop a laboratory instrument that is able to guarantee accurate virus classification in a shorter time.

This technique could also be applied to the analysis of biological agents in different matrices, such as in food, the perishability of which is a key factor for marketing. For instance, the consumption of raw or undercooked bivalve molluscan shellfish is the principal vehicle of Hepatitis A virus (e.g., in 2018, these shellfish were associated with 43.9% of Hepatitis A cases in Italy, where the incidence rate was 1.5 cases per 100.000 inhabitants^27^).

Moreover, this approach may find interest in clinical applications. Even in these cases, the virus isolation is quite long, providing an important response after days or weeks. Thus, when the time is shortened, important advantages may be obtained. However, a clinical application must be deeply investigated, as the presence of background material (e.g., proteins in blood) may be dominant, making the virus fluorescence signal fully covered by the background fluorescence. Thus, a pre-analytical phase may be needed. Regarding sensitivity, it can be tuned as mentioned in the discussion section (exposure time, radiation energy and collecting system improvements).

## Methods

### Fluorescence system

**Figure 5:**
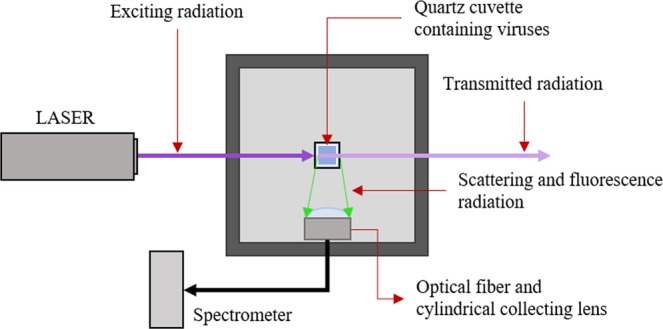
Experimental apparatus scheme.

The laser-induced fluorescence is based on the fluorophore excitation by electromagnetic radiation. Generally, the radiation used to induce the fluorescence is near-UV or UV light, whereas the fluorescence radiation is in the visible region. The light source used is a 266 nm Q-switched diode pumped solid-state laser (ELFORLIGHT – SPOT-10-20-266). The pulse frequency is 10 kHz, and the average power is 25 mW. The laser radiation passes through the cuvette where the virus sample is contained. The cuvette is made out of quartz since it is transparent to UV and visible radiation. A fibre optic is placed near the cuvette at 90° with respect to the laser beam. The optical fibre collects the fluorescence (and scattering) radiation and directs the collected light to the spectrometer. The spectrometer is an Ocean Optics USB2000 ranging from 350 nm to 1000 nm, with the CCD array composed of 2048 detectors (Sony ILX511B). The exposure time of the spectrometer ranges from 1 ms to 65 s. The cuvette and the collecting system are placed inside a closed box, which allows the exclusion of external radiation to reduce the background signal (sun, lamps, etc.). Two holes permit the laser beam to pass through the box and irradiate the sample. No optical filters are used to exclude scattering radiation. In fact, the first and the fourth harmonics are not recorded by the spectrometer, whereas the scattering of the second harmonic is sufficiently small to be filtered numerically (see data analysis section). Figure 5 shows the scheme of the experimental apparatus.

### Sample preparation and titration

The viruses included Coxsackie B4 (Enterovirus B, 10^7.5^ TCID50/mL), Coxsackie A7 (Enterovirus A, 10^6.5^ TCID50/mL), Coxsackie A9 (Enterovirus B, 10^7.2^ TCID50/mL), Hepatitis A (6.4 10^6^ genome copies), Echovirus (10^2^ TCID50/mL), Rotavirus (10^6^ TCID50/mL), and Astrovirus (human stool). The viruses were titrated according to the different cell lines, and, in particular, Coxsackie B4, A7, and A9 and Echovirus type 1 were titrated by the TCID50 method using a 96-well plate. Each well was seeded with 1 10^5^ cells and 100 µL of serial dilution of the virus; eight wells per dilution were used. The plates were incubated at 37 °C in 5% CO_2_ and checked each day for three days. The titration of Coxsackie and Echovirus type 1 was performed according to the Reed and Muench method^28^. The genome copies of Hepatitis A were calculated by qRT/PCR. Finally, the Astrovirus was obtained from a human case.

For each test, 1 mL of different viruses was concentrated in a Beckman Optima TL ultracentrifuge at 30,000 rpm/min/4 °C using a TLA-100.4 fixed angle rotor. The pellet was resuspended in 1 mL of Dulbecco completed PBS (Sigma D8537 DPBS). For the analysis, two additional mL of DPBS were added in the quartz cuvettes for each virus to achieve a 3.0 mL final volume.

### Experimental protocol

Two sets of measurements are performed to exclude random uncertainties from classification. Each set provides five spectra for each virus. Before each measurement, the background spectrum is acquired and removed from the fluorescence spectrum automatically. The background spectra are acquired by turning off the laser. The exposure time is always 60 s for background and fluorescence measurements.

### Data analysis

**Figure 6:**
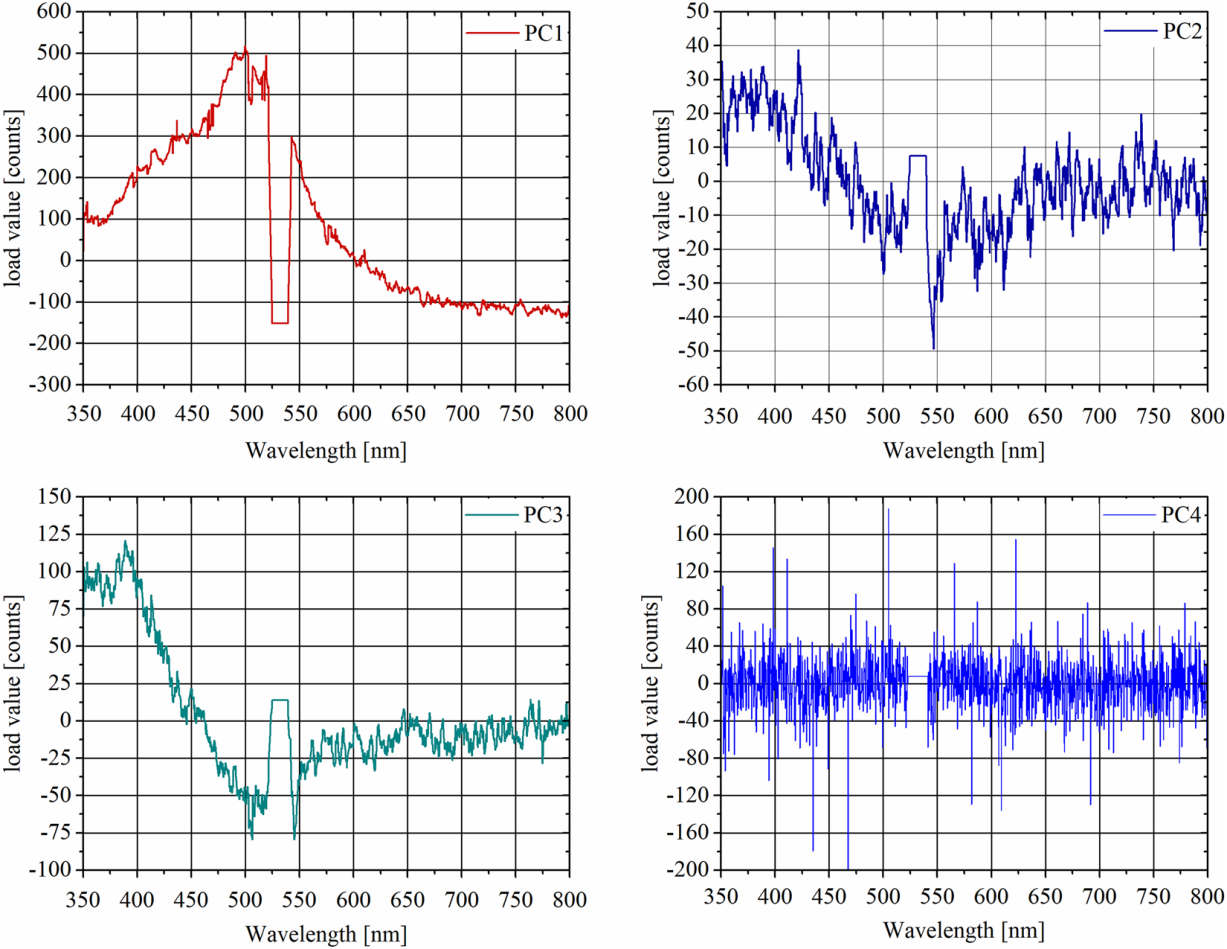
Loads of the first four principal components.

The spectra are pre-processed before the classification. First, the scattering peak at 532 nm (second harmonic) is removed. Then, a moving average filter is applied to reduce random noise. However, the classification is also performed without the latter filter, and the results show no significant differences. Principal component analysis (PCA) is applied before the use of the classification algorithms. PCA is commonly used to transform a set of observations with correlated variables in a set of linearly uncorrelated variables (principal components). This method has important advantages. First, it reduces the dimensions of the dataset. Furthermore, the first principal components are usually the ones that allow a good classification, as they explain most of the dataset variance. Thus, PCA reduces the efforts that the classification algorithms need to make to classify the data accurately. Figure 6 shows the loads of the first four principal components. The first three loads have specific trends as a function of the wavelength, meaning that the relative PCs are described by a specific linear combination of the entire wavelength range. The first gives more importance to the near-UV-visible region (350 nm to 520 nm), while the second and the third PCs highlight the features in the near-UV region. The fourth load shows an irrelevant trend, as it is only noise. In fact, the fourth PC, together with all the other higher-order PCs, is useless for classification. This means that most of the variability used for virus classification is described by the first three PCs, while the other ones are only noise.

The classification algorithms used are the decision tree, the support vector machine and the artificial neural network. The decision tree is a technique based on creating a list of decisions that allow the various classes to be distinguished, while the support vector machine creates a probabilistic function that is able to separate the classes. The SVM supports different kernels, which are probabilistic function models. They may be of any kind, such as linear, polynomial, or Gaussian. In this work, both polynomial (2^nd^ order) and Gaussian have been used, and both yielded the same results. The validation method for the decision tree and support vector machine is the leave-one-out cross-validation approach. The artificial neural networks are based on creating a net compounded by layers that contain a specific number of neurons. Neurons combine data inputs with specific weights. The training of the neural networks consists of assigning those weights to each input. The neural network used in this work is compounded by two layers: one hidden layer with a size of ten neurons and one output layer with six neurons (one for each class). To avoid overfitting, the spectral data are divided into three sets. Forty percent of the data are used for the training set, 35% for the validation set and 25% for the test set. Since the three sets are chosen randomly (guaranteeing that one spectrum of each class is always in each set), the neural network training and testing are performed thirty times to achieve a statistical result. The neural network is tested with and without PCA. In each case, the result is 100%.
